# Toppar: an interactive browser for viewing association study results

**DOI:** 10.1093/bioinformatics/btx840

**Published:** 2018-01-08

**Authors:** Thorhildur Juliusdottir, Karina Banasik, Neil R Robertson, Richard Mott, Mark I McCarthy

**Affiliations:** 1 Wellcome Trust Centre for Human Genetics, University of Oxford, Oxford, UK; 2 Novo Nordisk Center for Protein Research, University of Copenhagen, Copenhagen N, Denmark; 3 Oxford Centre for Diabetes, Endocrinology and Metabolism, University of Oxford, Churchill Hospital, Headington, Oxford, UK; 4 UCL Genetics Institute, University College London, London, UK; 5 Oxford University Hospitals Trust, Oxford NIHR Biomedical Research Centre, Churchill Hospital, Headington, Oxford, UK

## Abstract

**Summary:**

Data integration and visualization help geneticists make sense of large amounts of data. To help facilitate interpretation of genetic association data we developed Toppar, a customizable visualization tool that stores results from association studies and enables browsing over multiple results, by combining features from existing tools and linking to appropriate external databases.

**Availability and implementation:**

Detailed information on Toppar’s features and functionality are on our website http://mccarthy.well.ox.ac.uk/toppar/docs along with instructions on how to download, install and run Toppar. Our online version of Toppar is accessible from the website and can be test-driven using Firefox, Safari or Chrome on sub-sets of publicly available genome-wide association study anthropometric waist and body mass index data (Locke *et al.*, 2015; Shungin *et al.*, 2015) from the Genetic Investigation of ANthropometric Traits consortium.

## 1 Introduction

Analytical challenges facing genetic studies increase with larger, more complex datasets, more extensive phenotypic trait information, a greater array of statistical tests and a variety of genetic reference panels and annotation tools. Here, we present Toppar a customizable database-driven browser for genetic association genome-wide association study (GWAS) data. It allows integration and visualization of analyses generated across multiple platforms and methodologies. It combines a whole-genome overview of GWAS results with an interactive regional display for loci of interest. User data can be uploaded and explored in conjunction with gene and exon annotation, as well as already published genome-wide association catalogs (Welter *et al.*, 2014). The Toppar browser can filter its display using user-defined sub-categories, e.g. to visualize both single variant and gene-based association results. This enables comparison across multiple traits, using different tests and filters.

## 2 Implementation

### 2.1 Main features and functionality

**Fig. 1.**
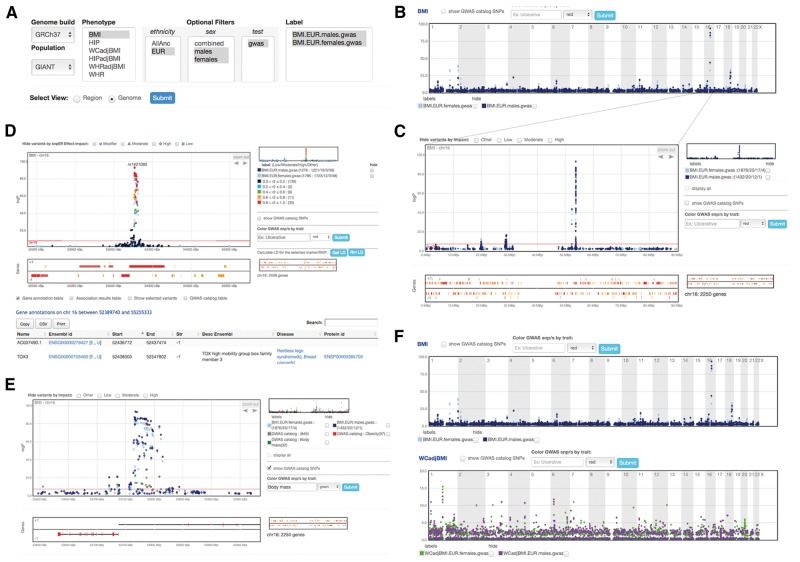
Toppar’s main interface. The filtering panel (**A**) allows the user to navigate and view the data by selecting available sub-categories. From the genome view (**B**), one navigates to a specific genomic locus by selecting it on the plot. This refocuses the display to the region (chromosome) view (**C**). Beyond a certain level of zoom-in the appearance of the plotted dots that indicate association results changes from small filled circles (**D**) to larger open circles, indicating they have become interactive and that test statistic associated with the point/test are displayed on mouseover. An interactive point becomes red when selected, with the variant name displayed above it, and the LD pattern of all data points relative to the one selected can be displayed (D). At further zoom-in, exonic regions are also displayed for each gene (**E**). GWAS catalog SNPs can be added and colored based on traits of interest and hovering over a GWAS variant displays its *rs* identifier, *P*-value and associated disease trait and pubmed id, while selecting the variant takes the user to the referenced pubmed article (E). Multiple traits can be viewed simultaneously (**F**), where each trait appears on a separate but synchronized plot: zooming in on one plot automatically refocuses the other plots

Toppar is an extension of GSCANDB (Taylor *et al.*, 2007), and the display includes filtering and interactive viewing panels (Fig. 1). Association results can be viewed in the latter by either genome or region view, with association *P*-values on the –log10 scale displayed on the vertical axes and chromosomal position on the horizontal axis. Association results mapped to a specific genome build (GRCh38 by default) for a given study or population can be viewed through various filters.

Toppar’s functionality is best illustrated using published genotype/phenotype association results, such as the waist and body mass index (BMI) datasets released by the international Genetic Investigation of ANthropometric Trait (GIANT) consortium, which includes GWAS data from a number of anthropometric traits stratified by ancestry and gender (Locke *et al.*, 2015; Shungin *et al.*, 2015). We uploaded sets of top 40 000 genetic variants associated with BMI and with traits related to fat distribution including waist-hip-ratio, with and without adjusting for BMI, to Toppar’s local database (see full instructions on website). The upload time per 40k set was around 3 s. The entire upload, which only has to be done once, took approximately 3 min on a Mac OS v10.9.5 with a 3 GHz Intel Core i7 processor and 16 GB 1600 MHz memory.

Once uploaded, the different phenotypes, ethnicity, gender and test appear in Toppar’s four filtering menus (Fig. 1A). To view the GIANT results (e.g. for BMI) one can use the optional filters before selecting genome view and pressing submit. From the genome view (Fig. 1B) one can navigate to the region (chromosome) view (Fig. 1C), where results for the area of interest and neighboring genes can be further explored through zooming and panning and linkage disequilibrium (LD) patterns can be displayed for selected variants. Genes linked with disease according to the DISEASE web resource, based on automatic text mining of scientific literature (Pletscher-Frankild *et al.*, 2015) are highlighted in red and the gene name and associated disease are displayed by hovering over the gene (Fig. 1C and D). Annotation for displayed genes, including gene name, links to Ensembl and UCSC, genomic interval and gene description appears in a table below the gene plot (as in Fig. 1D). Association results and GWAS catalog data are also listed in tables that are hidden from view by default.

Existing browsers that display genotype/phenotype association results include the regional plotter Locuszoom (Pruim *et al.*, 2010), UK10K genome browser (Geihs *et al.*, 2015) which is based on the Biodalliance platform (Down *et al.*, 2011) and the AMP T2D knowledge portal (http://www.type2diabetesgenetics.org). Locuszoom is optimized for plotting regions of association test results for single traits and displaying the pattern of LD, whereas the UK10K genome and AMP T2D are publicly available web browsers, which allow for the extensive exploration of pre-uploaded data. Toppar has the ability to display multiple trait data and integrate it with a wider selection of relevant external data as well as provide LD information for small genetic regions (GRCh38). It thus complements Locuszoom as well as larger genome browsers.

### 2.2 Installation and usage

Toppar was written in JavaScript and HTML and uses the Flot library (http://www.flotcharts.org) for plotting, zooming and panning. All data displayed on the plots are also listed in DataTables (https://datatables.net). Toppar stores the data in a MySQL database and uses Perl and DBI (Database Interface) with Common Gateway Interface for database communication. Installation of Toppar is straightforward, requiring an Internet-connected UNIX platform with a webserver (e.g. Apache) and Perl (required JavaScripts are included in the Toppar download package). The package also includes a Perl script for managing all database-dependent tasks, such as creating the database, and uploading and deleting data from it. The same script can also be used to update the database’s GWAS catalog and gene annotations making it straightforward to keep up with the most current releases. Data can also be uploaded and deleted through the online GUI.

The data format required for upload of association data is a text file containing at least four columns (chr, pos, marker_id and pvalue), where the exact order and naming of the columns is flexible (i.e. the chr label can be either chr, chrom or chromosome and is case insensitive) to suit a variety of output formats. Alternatively, the data can be uploaded from two separate files, one with variant information (chr, pos and marker_id) and the other with association results (marker_id and pvalue). Gene-based test results can also be directly uploaded to the database if the group file used to generate them is provided (see website for more information).

## 3 Conclusions

We have created a user-friendly tool to view and navigate phenotype/genotype association results. A distinctive feature of Toppar is that it facilitates navigation through results using a hierarchical filtering menu. Toppar is optimized for comparison of results obtained in bulk and immediately after receiving association data the user can view and browse the results. The user can store all data in one place, revisit it later, and add more results or external data to it as relevant. Thus, a key application of Toppar is that it can serve as a persistent local interface for association results and simultaneously integrate information from other sources for comparison and annotation. Future development of Toppar will focus on inclusion and display of regulatory elements [e.g. enhancers, promoters and topologically associating domains].

## Funding

This work was supported by the Wellcome Trust awards [090532 to R.M. and M.I.M, 098381] and the JDRF [2-SRA-2014-276-Q-R]. M.I.M is a Wellcome Trust Senior Investigator.

*Conflict of Interest*: none declared.
